# Genotype distribution of human papillomavirus (HPV) in histological sections of cervical intraepithelial neoplasia and invasive cervical carcinoma in Madrid, Spain

**DOI:** 10.1186/1471-2407-12-533

**Published:** 2012-11-20

**Authors:** Benjamín García-Espinosa, Ernesto Moro-Rodríguez, Emilio Álvarez-Fernández

**Affiliations:** 1 Department of Histology and Anatomical Pathology, Rey Juan Carlos University School of Medicine, Madrid, Spain; 2 Department of Anatomical Pathology and Laboratories, Hospital General Universitario “Gregorio Marañón”, Madrid, Spain; 3 Universidad Rey Juan Carlos, Av de Atenas s/n., E28922 Alcorcón, Madrid, Spain

**Keywords:** Human papillomavirus, Polymerase chain reaction, Genotyping, Squamous intraepithelial lesions, Cervix, Spain

## Abstract

**Background:**

Human Papillomavirus (HPV) genotype distribution and co-infection occurrence was studied in cervical specimens from the city of Madrid (Spain), as a contribution to the knowledge of Human Papillomavirus genotype distribution and prevalence of carcinogenic HPV types in cervical lesions in Spain.

**Methods:**

A total of 533 abnormal specimens, from the Hospital General Universitario “Gregorio Marañón” of Madrid, were studied. These included 19 benign lesions, 349 cervical intraepithelial neoplasias 1 (CIN1), 158 CIN2-3 and 7 invasive cervical carcinomas (ICC). HPV genotyping was performed using PCR and tube array hybridization.

**Results:**

We detected 20 different HPV types: 13 carcinogenic high-risk HPV types (HR-HPVs), 2 probably carcinogenic high-risk HPV types (PHR-HPVs) and 5 carcinogenic low-risk HPV types (LR-HPVs). The most frequent HPV genotypes found in all specimens were HPV16 (26.0%), 31 (10.7%) and 58 (8.0%). HPV 18 was only detected in 5.0%. Co-infections were found in 30.7% of CIN 1 and 18.4% cases of CIN2-3. The highest percentage of HR HPVs was found in those specimens with a CIN2-3 lesion (93.7%).

**Conclusion:**

As our study shows the current tetravalent vaccine could be effective in our geographical area for preventing all the invasive cervical carcinomas. In addition, upon the estimates of the important presence of other HR-HPV types – such as 31, 58, 33 and 52 – in different preneoplasic lesions the effectiveness of HPV vaccination in our geographical area, and others with similar genotype distribution, should be limited.

## Background

Cervical cancer is the second most common cancer in women worldwide. Several epidemiological studies make it possible to conclude that persistent infection by certain types of HPV is a causal and necessary factor for the development of cervical cancer. A series of HPV-induced precursor lesions, from LSIL to HSIL, may lead to ICC. More than 40 anogenital HPV types exist [1]. From an epidemiological point of view, based on their association with cervical cancer and precursor lesions, HPVs have been classified in two groups: high risk of carcinogenesis HPV types (HR-HPVs) and low risk of carcinogenesis HPV types (LR-HPVs) [2,3]. There are also two additional groups of probable high risk of carcinogenesis HPV types (PHR-HPVs) and indeterminate risk of carcinogenesis HPV (IR-HPVs) [4]. But even amongst the HR-HPV genotypes, the variation of their oncogenic potential is considerable, which may vary depending on specific intratypic HPV variations or on ethnic background and lifestyle factors of the human population under study.

HR-HPV 16 is the most common type in all studies, but there are some differences regarding its prevalence to other HPV types. HR-HPV 16 and 18 are estimated responsible for nearly 70% of cervical cancer cases worldwide. Co-infection with multiple HPV types could also increase the risk of cervical lesion. However, follow-up studies suggest that the presence of multiple types does not influence the course of HPV infections [5].

Two types of prophylactic vaccines have been developed to prevent cervical cancer: a bivalent vaccine against HR-HPV 16 and 18, and a tetravalent vaccine against HR-HPV 16/18 and LR-HPV 6 and 11 genotypes that are responsible for most genital warts [6-8].

Some studies suggest that these vaccines seem to protect against some HPV 16-related types (31, 33, 35 and 52) and HPV 18-related types (39, 45, 59, 68 and 85). However, vaccination against HPV 16/18 does not seem to cross-protect against HPV 58 [9,10]. In addition, the decreasing prevalence of the genotypes included in the HPV vaccine may increase the prevalence of other coinfecting genotypes. This hypothesis suggests that the elimination of certain genotypes by vaccination may affect the distribution of other genotypes, and the impact of the vaccine could vary [9].

Moreover, it has been demonstrated that the distribution of HPV 16 and 18 and the other HR-HPV genotypes vary around the world both in type and relative incidence. Therefore, the effect of HPV16/18 vaccines will, at least to some degree, vary by region. However, regional differences appear to become less pronounced with increasing severity of lesions, as HPV 16 becomes increasingly dominant [11].

The age-adjusted HPV prevalence in Spanish women from general population is very low (3%) [12]. However, this prevalence increase to 34.4% in Spanish women with cytological alterations [13]. In addition, changes in sexual behaviour and human migration flows could contribute to introduce some degree of variation both in prevalence and distribution of HPV types [14]. Additional genotypes that are not included in the first generation of human papillomavirus vaccines are frequently associated with cervical cancer in Latin America and Africa [15]. Over the past decade a large number of immigrants have arrived in Spain; more or less 40% come from Latin America and 50% are women, and the prevalence of HR-HPVs is more than three times higher in Latin Americans than in Spaniards [14]. Therefore, this significant immigration from Latin America, as well as from Eastern Europe and North Africa, possibly leads to the appearance in Spain of additional carcinogenic genotypes not included in current HPV vaccines [16,17].

For all these reasons, it is important to estimate the prevalence of different HPV types found in cervical cancer in different geographic regions and over time in order to study the carcinogenic function of each genotype, to assess the impact of the current vaccines and to guide the introduction of a new generation of them [13,18,19].

The regional community of Madrid is located in the centre of Spain and has a population of 6.4 million. In the last ten years, a high influx of immigrants from Latin America, Eastern Europe and North Africa has caused a population increase of nearly 15 per cent. The objective of this study is to provide epidemiological data regarding the prevalence and distribution of different HPV genotypes in samples with different grade of cervical lesions, obtained in our geographical area, in order to understand the carcinogenic potential of each of HPV type found and to discern the impact of HPV vaccination on our population.

## Methods

### Specimen collection and diagnosis

This is a cross-sectional and retrospective study, in which all cases diagnosed between January 2005 and July 2011 (1,137 abnormal specimens from fixed tissue sections of biopsies and LEEPs) were selected. Samples for HPV testing, containing cervical carcinoma related lesions, were collected by different departments of the Hospital General Universitario “Gregorio Marañón” of Madrid and submitted to the anatomical pathology laboratory of this hospital. In 533 of this specimens one or several HPV types was detected. The population included in this study had been selected for any type of gynaecologic pathology. This hospital provides healthcare to a population of about 750,000 individuals (11.5% of the population of the regional community of Madrid).

The positive samples included 19 benign lesions (genital warts, condylomas and papillomas), 349 cervical intraepithelial neoplasias 1 (CIN1), 158 CIN2-3 and 7 invasive cervical carcinomas (ICC).

Informed consent was not required for this study since the results presented here come from HPV genotyping routinely performed, as an adjunct to the cytological and histological study, in an anatomical pathology laboratory. The detection and genotyping was done in clinical setting and in order to protect patient confidentiality the identifiers of personal data were always deleted, that is why it was not possible to determine the ethnic of each patient. The study was supervised by the ethical committee of our hospital (Comité ético de investigación clínica - CEIC).

### Detection and genotyping of HPV

The DNA was obtained from fixed tissue sections of buffered formalin and embedded in paraffin (Kit for DNA extraction. Master Diagnostica SL Granada).

This DNA was used as a template to detect the presence of HPV DNA by PCR amplification using primers specific GP5-6 L1 consensus region (HPV screening kit. Master Diagnostica SL Granada).

The result of the amplification was visualized by agarose gel electrophoresis and ethidium bromide staining on a UV transilluminator, considering the case as positive if it showed a band of 150 bp.

Among the HPV positive cases we identified the type of virus using a kit that allows the detection of 14 specific HPV types of high-risk (16, 18, 31, 33, 35, 39, 45, 51, 52, 56, 58, 59, 68), 2 HPV types of intermediate-risk (53, 66) of and 5 HPV types of low-risk (6, 11, 42, 43, 44). This identification was performed by PCR amplification of a fragment of 450 bp L1 consensus region and reverse hybridization with probes specific for each type (HPV GenoArray Test Kit. Ref: IHP301-C. Master Diagnostica SL Granada. Supplier: HybriBio Limited, Hong Kong).

Two methods were used to estimate the frequency of HPV positivity: percentages referred to the number of lesions infected by one or several genotypes and percentages referred to the total number of virus detected in each kind of lesion and in the total of them.

### Statistical analysis

Statistical analysis was performed using Stata version 11.1/SE (StataCorp. LP, TX, USA).

Relative frequencies of HPV genotypes were estimated as percentages and their 95% confidence intervals were obtained with Clopper-Pearson method based on exact binomial distribution of tail areas.

## Results

### Distribution of viral genotypes

The percentage of HPV positivity in the total of specimens studied was at less than 47% (533/1,137).

Taking into account overall data, we detected 20 different HPV types: 13 HR-HPVs, 2 PHR-HPVs and 5 LR-HPVs.

HPV 16 was the most common type (26.0%) followed, in order of decreasing frequency, by HPV 31 (10.7%), HPV 58 (8.0%), HPV 6 (6.2%), HPV 33 (6.1%), HPV 52 (5.7%), HPV 53 (5.1%), HPV 18 (5.0%), HPV 66 (4.4%) and HPV 11 (4.3%).

HPV 16 was present in 35.3% of total lesions, HPV 31 in 14.4%, HPV 58 in 10.9%, HPV 6 in 8.4% and HPV 33 in 8.3% of them.

In the whole of lesions included in our study, 63.0% of HPVs detected were HR-HPVs, 12.2% were PHR-HPVs and 15.2% were LR-HPVs. HPVs 16 and/or 18 were founded in 41.3% of lesions and HPVs 6 and 11 in 14.1% of them.

Nine (1.2%) of HPV-positive cases detected were classified as an uncharacterized type (HPV X), but it is likely that this classification was related with a missed detection of known types and was not associated to an infection by HPV types to be discovered (Table 1).

**Table 1:** Frequency of HPV positivity according to the type of cervical lesion found

**Pathological diagnosis**	**Number (%) HPV + cases**	**Number (%) single HPV cases**	**Number (%) multiple HPV cases**	**Number (%) HPV X cases**
**Benign lesions**	19 (3.6%)	19 (100%)	0 (0%)	1 (5.3%)
**CIN 1**	349 (65.5%)	242 (69.3%)	107 (30.7%)	5 (1.4%)
**CIN 2-3**	158 (29.6%)	129 (81.6%)	29 (18.4%)	3 (1.9%)
**ICC**	7 (1.3%)	7 (100%)	0 (0%)	0 (0%)
**Total cases**	533 (100%)	397 (74.5%)	136 (25.5%)	9 (1.7%)

### Relationship between diagnoses and HPV genotypes

*In benign lesions*, HPV 6 was the most common type (42.1%) followed, in order of decreasing frequency, by HPV 11 (26.3%), HPV 16 (15.8%), HPV 18 (5.3%) and HPV 53 (5.3%).

21.1% of HPVs detected were HR-HPVs, 5.3% were PHR-HPVs and 68.4% were LR-HPVs. HPVs 16 and/or 18 comprised 21.1% of HPV infections detected and HPVs 6 and/or 11 comprised 68.4% of them. Therefore, LR-HPV types included in tetravalent vaccine were detected more frequently than viruses not included in this vaccine.

*In CIN1 cases*, HPV 16 was the most common type (18.2%) followed, in order of decreasing frequency, by HPV 31 (12.0%), HPV 58 (9.0%), HPV 6 (6.4%), HPV 53 (6.4%), HPV 33 (6.0%), HPV 66 (6.0%), HPV 52 (5.2%), HPV 18 (5.0%) and HPV 68 (4.8%).

HPV 16 was present in 26.1% of CIN1 lesions, HPV 31 in 17.2%, HPV 58 in 12.9%, HPV 53 in 9.2% and HPV 6 in 9.2% of them.

HR-HPVs were detected in 51.0% of CIN1 lesions, PHR-HPVs in 16.6% and LR-HPVs in 16.3% of them. HPVs 16 and/or 18 were present in 32.4% of lesions and HPVs 6 and/or 11 in 14.6% of them.

HR-HPV types not included in vaccines were detected more frequently (59.9%) than carcinogenic viruses included in vaccines (32.4%). LR-HPV types were infrequently identified as single infections (15.3%).

*In CIN2*-*3 cases*, HPV 16 was the most common type (45.2%) followed, in order of decreasing frequency, by HPV 31 (8.6%), HPV 52 (7.6%), HPV 33 (7.1%), HPV 58 (6.6%), HPV 18 (4.6%), HPV 6 (2.5%), HPV 11 (2.5%), HPV 51 (2.5%), HPV 53 (7.1%) and HPV 68 (2.0%).

HPV 16 was present in 56.3% of CIN2-3 lesions, HPV 31 in 10.8%, HPV 52 in 9.5%, HPV 33 in 8.9% and HPV 58 in 8.2% of them.

HR-HPVs were detected in 93.7% of CIN2-3 lesions, PHR-HPVs in 3.8% and LR-HPVs in 6.3% of them. HPVs 16 and/or 18 were present in 61.4% of lesions and HPVs 6 and/or 11 in 6.3% of them.

HR-HPV types not included in vaccines were detected with an inferior frequency (42.4%) to carcinogenic viruses included in vaccines (61.4%). LR-HPV types were rarely identified as single infections (4.7%).

*In ICC cases*, HPV 16 was the most common type (71.4%) followed, in order of decreasing frequency, by HPV 18 (14.3%) and HPV 11 (14.3%).

HR-HPVs were detected in 85.7% of ICC lesions and LR-HPVs in 14.3% of them. PHR-HPVs were not detected in this kind of lesions. HPVs 16 and/or 18 were present in 85.7% of lesions and HPVs 6 and/or 11 in 14.3% of them.

The distribution of HPV types and the analysis between pathological groups vs. HPV risk types is shown in Tables 2 and 3.

**Table 2:** Distribution of HPV genotypes found in the study according to the pathological diagnosis

**Genotype found**	**Total lesions**					**Benign lesions**					**CIN 1**				
	**N**	% *	**CI95%**	% **	**CI95%**	**N**	% *	**CI95%**	% **	**CI95%**	**N**	% *	**CI95%**	% **	**CI95%**
**HR-HPVs**															
**16**	188	35.3	31.2-39.5	26.0	22.9-29.4	3	15.8	3.4-39.6	15.8	3.4-39.6	91	26.1	21.5-31.0	18.2	14.9-21.9
**18**	36	6.8	4.8-9.2	5.0	3.5-6.8	1	5.3	0.1-26.0	5.3	0.1-26.0	25	7.2	4.7-10.4	5.0	3.3-7.3
**31**	77	14.4	11.6-17.7	10.7	8.5-13.1	0	0	0.0-0.0	0	0.0-0.0	60	17.2	13.4-21.6	12.0	9.3-15.2
**33**	44	8.3	6.1-10.9	6.1	4.5-8.1	0	0	0.0-0.0	0	0.0-0.0	30	8.6	5.9-12.0	6.0	4.1-8.5
**35**	9	1.7	0.8-3.2	1.2	0.6-2.4	0	0	0.0-0.0	0	0.0-0.0	6	1.7	0.6-3.7	1.2	0.4-2.6
**39**	9	1.7	0.8-3.2	1.2	0.6-2.4	0	0	0.0-0.0	0	0.0-0.0	6	1.7	0.6-3.7	1.2	0.4-2.6
**45**	19	3.6	2.2-5.5	2.6	1.6-4.1	0	0	0.0-0.0	0	0.0-0.0	16	4.6	2.6-7.3	3.2	1.8-5.2
**51**	21	3.9	2.5-6.0	2.9	1.8-4.4	0	0	0.0-0.0	0	0.0-0.0	16	4.6	2.6-7.3	3.2	1.8-5.2
**52**	41	7.7	5.6-10.3	5.7	4.1-7.6	0	0	0.0-0.0	0	0.0-0.0	26	7.4	4.9-10.7	5.2	3.4-7.5
**56**	14	2.6	1.4-4.4	1.9	1.1-3.2	0	0	0.0-0.0	0	0.0-0.0	12	3.4	1.8-5.9	2.4	1.2-4.2
**58**	58	10.9	8.4-13.8	8.0	6.2-10.3	0	0	0.0-0.0	0	0.0-0.0	45	12.9	9.6-16.9	9.0	6.7-11.9
**59**	15	2.8	1.6-4.6	2.1	1.2-3.4	0	0	0.0-0.0	0	0.0-0.0	14	4.0	2.2-6.6	2.8	1.5-4.7
**68**	28	5.3	3.5-7.5	3.9	2.6-5.6	0	0	0.0-0.0	0	0.0-0.0	24	6.9	4.5-10.1	4.8	3.1-7.1
**PHR-HPVs**															
**53**	37	6.9	4.9-9.4	5.1	3.6-7.0	1	5.3	0.1-26.0	5.3	0.1-26.0	32	9.2	6.4-12.7	6.4	4.4-8.9
**66**	32	6.0	4.1-8.4	4.4	3.1-6.2	0	0	0.0-0.0	0	0.0-0.0	30	8.6	5.9-12.0	6.0	4.1-8.5
**LR-HPVs**															
**6**	45	8.4	6.2-11.1	6.2	4.6-8.3	8	42.1	20.3-66.5	42.1	20.3-66.5	32	9.2	6.4-12.7	6.4	4.4-8.9
**11**	31	5.8	4.0-8.2	4.3	2.9-6.0	5	26.3	9.1-51.2	26.3	9.1-51.2	20	5.7	3.5-8.7	4.0	2.5-6.1
**42**	3	0.6	0.1-1.6	0.4	0.1-1.2	0	0	0.0-0.0	0	0.0-0.0	3	0.9	0.2-2.5	0.6	0.1-1.7
**43**	1	0.2	0.0-1.0	0.1	0.0-0.8	0	0	0.0-0.0	0	0.0-0.0	1	0.3	0.0-1.6	0.2	0.0-1.1
**44**	5	0.9	0.3-2.2	0.7	0.2-1.6	0	0	0.0-0.0	0	0.0-0.0	5	1.4	0.5-3.3	1.0	0.3-2.3
**X**	9	1.7	0.8-3.2	1.2	0.6-2.4	1	5.3	0.1-26.0	5.3	0.1-26.0	5	1.4	0.5-3.3	1.0	0.3-2.3

N: total number of times which each genotype was detected.

* Percentages referred to the number of lesions infected by one or several genotypes (533 Total lesions, 19 benign lesions and 349 CIN 1).

** Percentages referred to the total number of virus detected (722 viruses in the Total of lesions, 19 in the Benign lesions and 499 in CIN 1).

CI95%: 95% confidence intervals used for estimate percentages.

**Table 3:** Distribution of HPV genotypes found in the study according to the pathological diagnosis

**Genotype found**	**CIN 2-3**					**Invasive carcinoma**				
	**N**	% *	**CI95%**	% **	**CI95%**	**N**	% *	**CI95%**	% **	**CI95%**
**HR-HPVs**										
**16**	89	56.3	48.2-64.2	45.2	38.1-52.4	5	71.4	29.0-96.3	71.4	29.0-96.3
**18**	9	5.7	2.6-10.5	4.6	2.1-8.5	1	14.3	0.4-57.9	14.3	0.4-57.9
**31**	17	10.8	6.4-16.7	8.6	5.1-13.5	0	0	0.0-0.0	0	0.0-0.0
**33**	14	8.9	4.9-14.4	7.1	3.9-11.6	0	0	0.0-0.0	0	0.0-0.0
**35**	3	1.9	0.4-5.4	1.5	0.3-4.4	0	0	0.0-0.0	0	0.0-0.0
**39**	3	1.9	0.4-5.4	1.5	0.3-4.4	0	0	0.0-0.0	0	0.0-0.0
**45**	3	1.9	0.4-5.4	1.5	0.3-4.4	0	0	0.0-0.0	0	0.0-0.0
**51**	5	3.2	1.0-7.2	2.5	0.8-5.8	0	0	0.0-0.0	0	0.0-0.0
**52**	15	9.5	5.4-15.2	7.6	4.3-12.2	0	0	0.0-0.0	0	0.0-0.0
**56**	2	1.3	0.2-4.5	1.0	0.1-3.6	0	0	0.0-0.0	0	0.0-0.0
**58**	13	8.2	4.5-13.7	6.6	3.6-11.0	0	0	0.0-0.0	0	0.0-0.0
**59**	1	0.6	0.0-3.5	0.5	0.0-2.8	0	0	0.0-0.0	0	0.0-0.0
**68**	4	2.5	0.7-6.4	2.0	0.6-5.1	0	0	0.0-0.0	0	0.0-0.0
**PHR-HPVs**										
**53**	4	2.5	0.7-6.4	2.0	0.6-5.1	0	0	0.0-0.0	0	0.0-0.0
**66**	2	1.3	0.2-4.5	1.0	0.1-3.6	0	0	0.0-0.0	0	0.0-0.0
**LR-HPVs**										
**6**	5	3.2	1.0-7.2	2.5	0.8-5.8	0	0	0.0-0.0	0	0.0-0.0
**11**	5	3.2	1.0-7.2	2.5	0.8-5.8	1	14.3	0.4-57.9	14.3	0.4-57.9
**42**	0	0	0.0-0.0	0	0.0-0.0	0	0	0.0-0.0	0	0.0-0.0
**43**	0	0	0.0-0.0	0	0.0-0.0	0	0	0.0-0.0	0	0.0-0.0
**44**	0	0	0.0-0.0	0	0.0-0.0	0	0	0.0-0.0	0	0.0-0.0
**X**	3	1.9	0.4-5.4	1.5	0.3-4.4	0	0	0.0-0.0	0	0.0-0.0

N: total number of times which each genotype was detected.

* Percentages referred to the number of lesions infected by one or several genotypes (158 CIN 2-3 and 7 invasive carcinomas).

** Percentages referred to the total number of virus detected (197 viruses in CIN 2-3 and 7 in Invasive carcinomas).

CI95%: 95% confidence intervals used for estimate percentages.

### Genotypes found as co-infections

The percentages of multiple infections were 30.7% in CIN1 cases and 18.4% in CIN2-3 cases. Multiple infections were not detected in benign lesions and in ICC.

In all sorts of lesions with multiple infections the most common pattern of co-infection was double infection with HPV 16 and 58 (6 cases), followed by double infection with HPV 16/31 (5 cases), HPV 16/53 (5 cases), HPV 16/18 (4 cases) and 16/52 (4 cases).

*In CIN1 multiple infections cases*, most co-infections consisted of two different genotypes (71.0%). 20.6% of the cases showed triple genotype infection and only 6.5% of them showed quadruple genotype infection. Surprisingly, in one case (0.9%) five different genotypes were found and in other case (0.9%) six different genotypes were found.

The most common pattern of co-infection was double infection with HPV 16 and 58 (5 cases). Other patterns of co-infection were: HPV 16/31 (4 cases), HPV 16/53 (4 cases), HPV 16/18 (3 cases), and HPV 31/6 (3 cases).

In this kind of multiple infections, HPV 16 was the most common type (16.3%) followed, in order of decreasing frequency, by HPV 31 (10.5%), HPV 66 (9.7%), HPV 58 (7.4%), HPV 68 (7.4%), HPV 53 (7.4%), HPV 33 (6.2%), HPV 52 (6.2%), HPV 18 (4.7%) and HPV 6 (4.7%).

HR-HPVs were detected in 96.3% of CIN1 lesions with co-infection, PHR-HPVs in 37.4% and LR-HPVs in 15.9% of them. HPVs 16 and/or 18 were present in 46.7% of this cases and HPVs 6 and/or 11 in 15.0% of them.

*In CIN2*-*3 multiple infections cases*, most co-infections consisted of two different genotypes (69.0%). 27.6% of cases showed triple genotype infection and only 3.4% of them showed quadruple genotype infection.

The most common pattern of co-infection was double infection with HPV 16 and 52 (3 cases) and triple infection with HPV 16, 33 and 58 (2 cases).

In this kind of multiple infections, HPV 16 was the most common type (23.5%) followed, in order of decreasing frequency, by HPV 33 (11.8%), HPV 52 (11.8%), HPV 58 (8.8%), HPV 31 (7.4%), HPV 68 (5.9%), HPV 18 (4.4%), HPV 45 (4.4%), HPV 53 (4.4%) and HPV 6 (4.4%).

HR-HPVs were detected in 100% of CIN2-3 lesions with co-infection, PHR-HPVs in 17.2% and LR-HPVs in 13.8% of them. HPVs 16 and/or 18 were present in 62.1% of this cases and HPVs 6 and/or 11 in 13.8% of them.

The data concerning co-infections are shown in Tables 4 and 5.

**Table 4:** Pathological diagnoses and co-infection occurrence

**Pathological diagnosis**	**2 HPV types**	**3 HPV types**	**4 HPV types**	**5 HPV types**	**6 HPV types**	**Total**
	**nº (%)**	**nº (%)**	**nº (%)**	**nº (%)**	**nº (%)**	**nº (%)**
**CIN 1**	76 (71.0%)	22 (20.6%)	7 (6.5%)	1 (0.9%)	1 (0.9%)	107 (78.7%)
**CIN 2-3**	20 (69.0%)	8 (27.6%)	1 (3.4%)	0 (0.0%)	0 (0.0%)	29 (21.3%)
**Total**	96 (70.6%)	30 (22.1%)	8 (5.9%)	1 (0.7%)	1 (0.7%)	136 (100%)

Percentage referred to total of co-infections (n = 136).

**Table 5:** HPV genotype distribution in coinfection cases

**Genotype found**	**CIN 1**					**CIN 2-3**				
	**N**	% *	**CI95%**	% **	**CI95%**	**N**	% *	**CI95%**	% **	**CI95%**
**HR-HPVs**										
**16**	42	39.3	30.0-49.2	16.3	12.0-21.4	16	55.2	35.7-73.6	23.5	14.1-35.4
**18**	12	11.2	5.9-18.8	4.7	2.4-8.0	3	10.3	2.2-27.4	4.4	0.9-12.4
**31**	27	25.2	17.3-34.6	10.5	7.0-14.9	5	17.2	5.8-35.8	7.4	2.4-16.3
**33**	16	15.0	8.8-23.1	6.2	3.6-9.9	8	27.6	12.7-47.2	11.8	5.2-21.9
**35**	1	0.9	0.0-5.1	0.4	0.0-2.1	2	6.9	0.8-22.8	2.9	0.4-10.2
**39**	3	2.8	0.6-8.0	1.2	0.2-3.4	1	3.4	0.1-17.8	1.5	0.0-7.9
**45**	11	10.3	5.2-17.7	4.3	2.2-7.5	3	10.3	2.2-27.4	4.4	0.9-12.4
**51**	11	10.3	5.2-17.7	4.3	2.2-7.5	1	3.4	0.1-17.8	1.5	0.0-7.9
**52**	16	15.0	8.8-23.1	6.2	3.6-9.9	8	27.6	12.7-47.2	11.8	5.2-21.9
**56**	6	5.6	2.1-11.8	2.3	0.9-5.0	1	3.4	0.1-17.8	1.5	0.0-7.9
**58**	19	17.8	11.0-26.3	7.4	4.5-11.3	6	20.7	8.0-39.7	8.8	3.3-18.2
**59**	6	5.6	2.1-11.8	2.3	0.9-5.0	1	3.4	0.1-17.8	1.5	0.0-7.9
**68**	19	17.8	11.0-26.3	7.4	4.5-11.3	4	13.8	3.9-31.7	5.9	1.6-14.4
**PHR-HPVs**										
**53**	19	17.8	11.0-26.3	7.4	4.5-11.3	3	10.3	2.2-27.4	4.4	0.9-12.4
**66**	25	23.4	15.7-32.5	9.7	6.4-14.0	2	6.9	0.8-22.8	2.9	0.4-10.2
**LR-HPVs**										
**6**	12	11.2	5.9-18.8	4.7	2.4-8.0	3	10.3	2.2-27.4	4.4	0.9-12.4
**11**	5	4.7	1.5-10.6	1.9	0.6-4.5	1	3.4	0.1-17.8	1.5	0.0-7.9
**42**	3	2.8	0.6-8.0	1.2	0.2-3.4	0	0	0.0-0.0	0	0.0-0.0
**43**	1	0.9	0.0-5.1	0.4	0.0-2.1	0	0	0.0-0.0	0	0.0-0.0
**44**	3	2.8	0.6-8.0	1.2	0.2-3.4	0	0	0.0-0.0	0	0.0-0.0
**X**	0	0	0.0-0.0	0	0.0-0.0	0	0	0.0-0.0	0	0.0-0.0

N: total number of times which each genotype was detected.

* Percentages referred to the number of lesions infected by one or several genotypes (107 CIN 1 and 29 CIN 2-3 lesions).

**Percentages referred to the total number of virus detected in each kind of lesion (257 virus in CIN 1 and 68 in CIN 2-3 lesions).

CI95%: 95% confidence intervals used for estimate percentages.

## Discussion

Of all the lesions, HPV 16 was the most frequent genotype. This finding is in accordance with many other studies carried out worldwide [20]. In our study, this genotype was present in 35.3% of total lesions. However, in a previous study carried in our hospital from 1993 to 2000, the HPV 16 presence in the total number of lesions was somewhat higher than at present (39%) [21]. Also, in a recent study carried in a southern region of Spain from 2006 to 2007, this presence was even lower than ours (21.2%) [17].

As expected, HPV16 presence increased in accordance with the grade of the lesion (15.8% in benign lesions, 26.1% in CIN1 cases, 56.3% in CIN2-3 cases and 71.4% in ICC).

HPV 31 was the second most frequent genotype in CIN1 lesions and in CIN2-3 lesions. In a study carried in an eastern region of Spain, HPV 31 was also the second most frequent genotype in HSIL lesions and the presence found in these (10.8%) was very similar to the presence obtained in our study (10.8%) [19]. Previous meta-analysis reported the second position of HPV 31 in LSILs in Europe [11,20], and our results confirm this finding.

After HPV 16 and 31, HPV 58 was the third most common genotype found in our study. This event should be explained by the large number of immigrants who arrived in Spain over the past decade from Latin America, where this genotype is the second most frequently detected in HSIL after HPV 16 [13,19]. However, additional studies are needed to confirm this hypothesis. Moreover, the HPV 58 presence was somewhat higher in CIN1 cases (12.9%) than in CIN2-3 cases (8.2%), but this finding doesn’t have statistical significance attending the 95% confidence intervals used for estimate these percentages (9.6-16.9 versus 4.5-13.7).

Our HPV 58 findings are in accordance with other recent study carried in a south-eastern region of Spain in which HPV 58 was the third most common genotype found and its frequency progressively decreased as lesions showed higher grades of dysplasia [22]. As some authors have reported, this decreasing HPV 58 frequency with the severity of the lesion may indicate that, despite the high frequency of HPV58 found in our area, LSIL caused by HPV58 would have less likelihood to progress to HSIL than a LSIL caused by HPV16 [22].

Globally, HPV 6 was the fourth most common genotype found in our study. Also, HPV 6 and 11 were the most frequent genotype in benign lesions (respectively, 42.1% and 26.3%); but, surprisingly, HPV 16 was present in third position (15.8%), as a single infection, in this type of lesions.

Some authors have reported that LR-HPV types -such as HPV 6 or HPV 11- are rarely identified as single infections in invasive cervical cancer [18]. In the previous study carried in our hospital, from 1993 to 2000, one case of single LR-HPV type infection in cervical cancer was reported [21]. Our current findings confirm that the detection of a single LR-HPV type (HPV 11) in ICC is a possible event. Further efforts are needed to understand in which conditions these HPV types can indeed induce invasive cervical cancer in rare circumstances.

In our study, the HPV 33 and 52 were the fifth and sixth most common types obtained. HPV 33 presence was basically no difference in CIN1 cases (8.6%) than in CIN2-3 cases (8.9%). This finding is in disagreement with the previously mentioned study carried in a south-eastern region of Spain in which HPV 33 frequency increased in parallel with the severity of the lesion [22].

One previous meta-analysis reported the seventh position of HPV 52 in HSIL in Europe [20], although in our study HPV 52 was third in these types of lesions. Therefore, the carcinogenic importance of this genotype is possibly increasing in our region at present.

Regarding the frequency of cases with the HPV 18 genotype is significant in our study that the frequency is not as high as the published in other international series. This data is in accordance with other published studies in Spain, in which HPV type 18 does not appear as a common type in the general population in our country [17]. Also, apparently, the HPV 18 presence was higher in CIN1 cases (7.2%) than in CIN2-3 cases (5.7%), but this finding doesn’t have statistical significance attending the 95% confidence intervals used for estimate these percentages (4.7-10.4 versus 2.6-10.5).

As expected, HPV 16 and 18 presence was appreciably lower in CIN1 cases (32.4%) than in CIN2-3 cases (61.4%). However, in a study carried in a north-eastern region of Spain from 1999 to 2005, the joint frequency of these genotypes was considerably higher (55% in CIN 1 cases, versus 80% in CIN 2 cases and 90% in CIN 3 cases) [16].

Moreover, in the earliest study carried in our hospital, from 1993 to 2000, the HPV 16/18 frequency was somewhat higher than in our current study (43% in LSIL and 67% en HSIL) [21]. However, in a later study also carried in our hospital, the frequency of 16/18 HPV types was similar to our present study (41% in all types of lesions) [23]. Therefore, the joint frequency of these genotypes and its carcinogenic importance is possibly decreasing over time in our region.

A relationship was found between lesions and HR-HPVs frequency. Thus, these genotypes were found more frequently in CIN2-3 cases (93.7%) than in CIN1 cases (51.0%) or benign lesions (21.1%). The HR-HPVs presence found in our study in CIN2-3 cases (93.7%) was similar to the frequency found in the previous studies carried in a region of northern Spain (88.1%) and in a region in eastern Spain (87.4%) [13,19].

Another issue worth mentioning is the co-infection occurrence, which seems to be more frequent in CIN1 cases (30.7%) than in CIN2-3 cases (18.4%). This finding is in accordance with the study carried in a south-eastern Spanish region mentioned above [22]; but it is at variance with the other previously mentioned study carried in a region in eastern Spain, in which the percentages of multiple infections were lower than in our study, and there were no considerable differences between groups of lesions (4.8% in LSIL and 3.7% in HSIL) [19].

In all types of lesions with multiple infections, the most common pattern of co-infection was double infection with HPV 16 and 58 (6 cases). The same result was found in the previously mentioned study carried in a region in northern Spain [13]. However, the most common pattern of co-infection in HSILs cases was double infection with HPV 16 y 52. It is unknown whether the association of these genotypes can produce a synergistic effect and increase carcinogenic risk. Further efforts are needed to clarify this hypothesis.

## Conclusion

Regional variations in prevalence and distribution of HPV types have been verified in several epidemiological studies. In some countries such as Spain, these changes could be due to the arrival of a large number of women immigrants over the past decade. Furthermore, the generalized introduction of a HPV vaccination could produce an epidemiological pattern change and, consequently, in the coming years the majority of HPV infections could be produced by HPV types different from 16 and 18.

As our study shows the current tetravalent vaccine could be effective in our geographical area for preventing all the invasive cervical carcinomas, however, the joint frequency of HPV 16/18 and its carcinogenic importance is possibly decreasing over time in our region. In addition, our study provides estimates of the important presence of other HR-HPV types – such as 31, 58, 33 and 52 – in different preneoplasic lesions. Thus, in the future the effectiveness of HPV vaccination in our geographical area and others with similar genotype distribution should be limited.

Therefore, the assessment of the distribution of HPV types associated with cervical cancer, in different geographic areas and over time, is necessary in order to apply the correct diagnostic and therapeutic measures in each region, to assess the impact of current vaccines and to guide the introduction of a new generation of them.

### Weaknesses of the study

Our study presents some limitations. First, the origin of the samples from only one hospital may do hard to generalize the results to the entire population of Spain. However, this hospital is a reference centre in which patients of many districts and municipalities of the Region of Madrid are attended. Also, the results obtained in this study agree with data about population located in other Spanish regions and previously published.

Second, whereas more than 40 anogenital HPV types exist, only 20 were detected in this study. But all the HPVs genotypes usually implicated in the origin of the cervical cancer are detected.

Third, the small sample size of benign lesions and ICC affect to the study’s significance. However, the difficulty to obtain ICC cases in Spain gives value to these results. In the future, it would be of interest to obtain more cases for further studies.

## Abbreviations

HPV: Human papillomavirus; HR-HPV: High risk of carcinogenesis HPV type; PHR-HPV: Probably high risk of carcinogenesis HPV type; LR-HPV: Low risk of carcinogenesis HPV type; ICC: Invasive cervical carcinoma; CIN: Cervical intraepithelial neoplasia.

## Competing interests

The authors declare that they have non-financial competing interests.

## Authors’ contributions

BGE participated in the design of the study, global data acquisition, analysis and preparation of the first draft. EMR revised critically the first and final draft, participated in the data acquisition, and conceived the design of the study, EAF conceptual and planning of the design of the study, surgical pathology data acquisition and analysis. All authors read and approved the final manuscript.

## Pre-publication history

The pre-publication history for this paper can be accessed here:

http://www.biomedcentral.com/1471-2407/12/533/prepub
